# Elite rock-climbers exhibit early signs of degenerative spinal changes; enhanced analysis using quantitative MRI

**DOI:** 10.1186/s13102-026-01579-y

**Published:** 2026-02-13

**Authors:** Henrik Hedelin, Fredrik Identeg, Alice Nilsson, Hanna Hebelka, Kerstin Lagerstrand

**Affiliations:** 1 Department of Orthopaedics, NU-Hospital Group, Region Västra Götaland, Trollhättan/Uddevalla, Sweden; 2 Department of Orthopaedics, Sahlgrenska University Hospital, Region Västra Götaland, Gothenburg, Sweden; 3 Institute of Clinical Sciences, Sahlgrenska Academy, University of Gothenburg, Gothenburg, Sweden; 4 Department of Radiology, Sahlgrenska University Hospital, Region Västra Götaland, Gothenburg, Sweden; 5 Department of Biomedical Engineering and Medical Physics, Sahlgrenska University Hospital, Region Västra Götaland, Gothenburg, Sweden

**Keywords:** Rock-climbing, Athletes, Intravertebral discs, Magnetic resonance imaging, Thoracic spine, Lumbar spine, Disc degeneration, Endplate, MRI biomarkers

## Abstract

**Background:**

Climbing has emerged as an increasingly popular competitive sport, leading to more demanding training regimens that often begin at a younger age. Competitive climbing training contains elements that, based on evidence from other sports, could predispose athletes to early degenerative changes in the spine. The one previous report that exists on this subject could not demonstrate any significant difference between elite climbers and controls. The present study employs objective quantitative measurements with the aim to detect early spinal changes in climbing athletes.

**Methods:**

Fifteen climbers consisting of the Swedish national team and a group training for selection to the national team [age mean (Standard Deviation, SD): 23.1(3.2) years; 7 men] were included. A control group, matched in age and sex [24.3(1.5) years; 7 men], were recruited.

Participants were scanned with a 1.5 T MRI system using standard spine protocols, including sagittal T1- and T2-weighted imaging covering Th1 to S1. Data-driven analysis of the T2-weighted images was performed to extract signal mean from midsagittal disc slices, both for the whole inner region and for subregions in the anterior to posterior direction.

**Results:**

Significant differences in disc signal intensity between climbers and controls were observed both globally and across specific subregions. The difference was most prominent at the thoracolumbar junction (*p*<0.05 in T10-L4 and *p*<0.01 T12-L2) with lower signal levels in the climbing group. In the subregion analysis, the central region, representing the nucleus pulposus, showed the greatest difference between groups.

**Conclusions:**

The present method identified early signs of disc degeneration in the thoracolumbar spine of elite level climbers compared to controls. The signal reduction, representing disc dehydration, was identified in multiple discs and was more pronounced in the thoracolumbar junction and in the central parts of the discs. As disc dehydration is a recognized risk factor for annular fissures and other degenerative changes, these findings may reflect early stress-related degeneration associated with high training loads in climbing athletes.

**Supplementary Information:**

The online version contains supplementary material available at 10.1186/s13102-026-01579-y.

## Introduction

Climbing, as a competitive sport, is a fairly new phenomenon and was admitted as an Olympic sport in 2020 [1]. Because of this increased popularity, rigorous training regimes are increasingly started at an adolescent age [2]. A key aspect of training for competitive climbing is the climbing discipline of bouldering, where the athlete climbs shorter difficult routes of 2–4 m and repeatedly jumps or falls on a padded mat. These jumps place high, and largely uncontrolled load on the spine numerous times during each training session.

 Ex vivo models have demonstrated that repetitive axial loads may accelerate the degenerative process in both the intervertebral discs and the endplates [3–5]. It is also well established that elite athletes in certain sport disciplines have a high risk of developing early degenerative spinal changes [6–11]. These changes include general disc degeneration, disc herniations, spondylolysis and spondylolisthesis as well as characteristic apophyseal ring injuries [12].

Back pain is also a frequent complaint among top athletes in general, especially after training progression [13, 14]. The etiology behind back pain is complex but early degenerative changes are believed to play a significant role [15, 16]. In the light of this pathophysiology and the risk of overloading, it is important to identify degenerative changes at an early stage. Among climbers, only a single study to date has used MRI (Magnetic Resonance Imaging) to examine spinal changes, and found no significant differences in the prevalence of degenerative changes between climbers and non-climbers [17]. The same study also reported a high prevalence of thoracolumbar back pain among climbers, highlighting the need for a more detailed investigation of the subject.

One reason that the causality between spinal abnormalities and morbidity in athletes has proven elusive may be that most studies are based on plain radiographs or gross MRI markers [8, 18–26] and do not use quantifiable methods that can objectively measure tissue changes on a continuous scale. Quantitative biomarkers that are sensitized to relevant changes in the spinal tissue may enable early detection, prior to the onset of manifest and permanent damage, and could consequently enhance the development of injury prevention strategies and the adaptation of training regimens.

Advanced analysis of MRI images utilizing data-driven assessment of disc characteristics can provide objective parameters of disc health [27–29]. The advantage of such approach entails the ability to detect not only general, but also distinct degenerative changes in the discs, like annular fissuring and remodeling of the nucleus pulposus [28, 30].

The aim of this study was to determine whether climbers show early signs of disc degeneration in the thoracolumbar spine using quantitative MRI biomarkers.

## Materials and methods

The study builds on a data set, previously analyzed using non-quantitative MRI methods [17].

### Participants

Of 19 invited climbing participants, 15 accepted participation and were included along with 15 controls. The climbing group (mean age 23.1 (SD 3.2) years, 7 men, 8 women) and the control group (mean age 24.3 (SD 1.5) years, 7 men, 8 women) were matched for age, sex, height, accepting a minor difference in weight (Table 1).

Inclusion criteria of the climbing participants were: age over 18 years and a climbing level of elite over the last 12 months as defined by the International Rock Climbing Research Association (IRCRA) [31–33]. Additionally, a minimum of five years of climbing and current or previous participation in recognized climbing competitions was required. All participants competed in both sport climbing and bouldering and all frequently used bouldering as a training method. For all participants bouldering and sport climbing were trained from the start of their climbing career. All members of the Swedish national sport climbing team were included (*n* = 8). To increase the study group the head coach suggested additional elite climbers, training to participate in the team (*n* = 11).

The control group was recruited through advertisement in social media. Any regular climbing or previous participation in any sport at a competitive or elite level led to exclusion. For all participants, exclusion criteria were prior spinal surgery or any contraindication to MRI. Apart from demographics, the maximum red point climbing level over the last 12 months (bouldering or sport climbing), as classified by the standard reporting of IRCRA, as well as training volume was collected through a questionnaire developed for this study (appendix 1). Training-related questions were administered identically for the control group and the climbers, with the exception that the control group reported general training volume rather than climbing-specific training.All demographic variables were obtained via participant self-report.

### MRI examination

Participants were scanned using a 1.5 T MRI system (Signa, GE Healthcare, Chicago, IL, USA). Sagittal T1- and T2-weighted images were acquired from Th1 to S1 using 12 slices. See previous analysis for scan protocol details [17].

### Image analysis

All MRI image analyses were data-driven and therefore independent on observer input. Regionalized intensity signal analysis of each disc was performed on the T2-weighted images, following the method described by Waldenberg et al. [28]. All discs and cerebrospinal fluid in the images were automatically segmented using the software TotalSpineSeg, a tool based on nnU-Net [34–36]. For disc segmentation, depending on whether the disc extended across an odd or even number of sagittal slices, only three or four midsagittal slices were included. All images were normalized to the mean signal intensity of the segmented cerebrospinal fluid in each image.

**Fig. 1:**
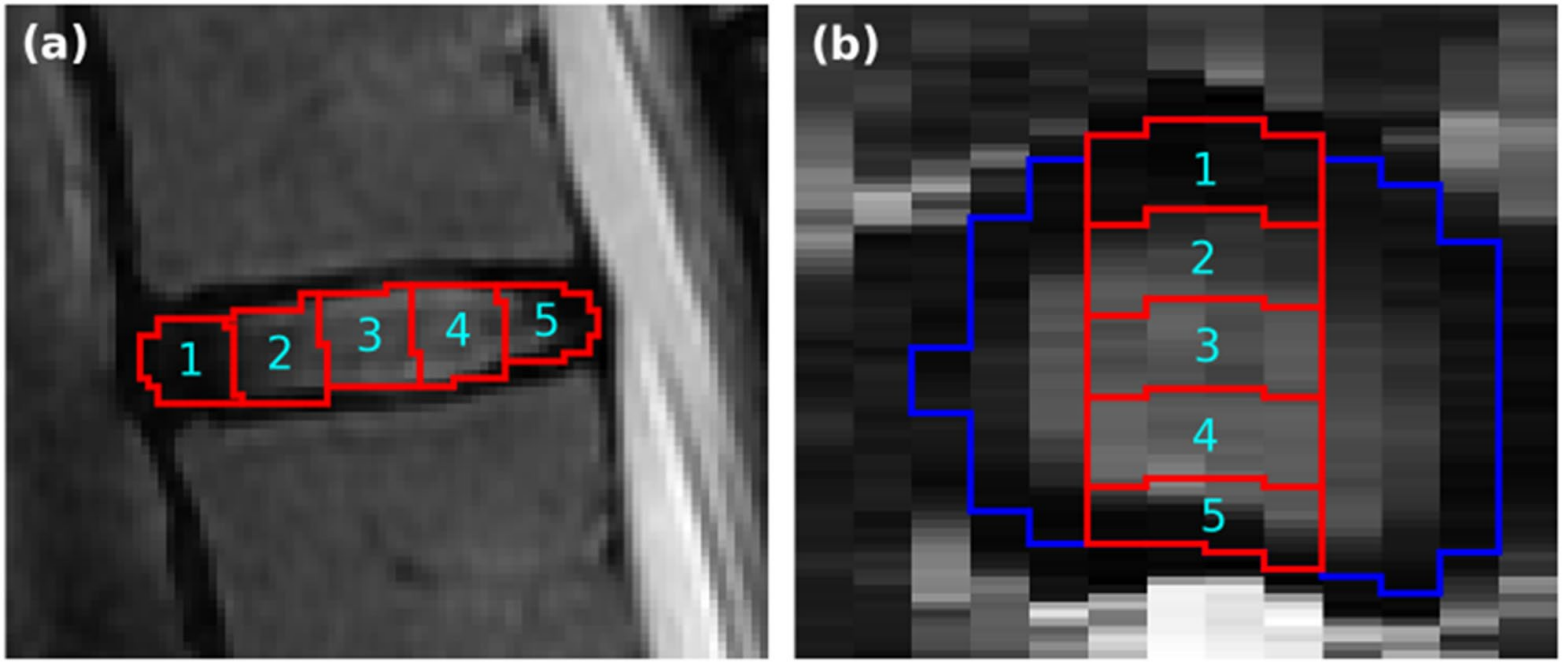
Segmentation and delineation of five subregions within a disc. (**a**) Sagittal view, displaying five subregions labelled from anterior (1) to posterior (5). (**b**) Axial view illustrating that the subregions are confined to the midsagittal slices. The blue delineation marks the border of the segmented disc

Subsequently, each segmented disc was automatically divided into subregions extending in the anterior-posterior direction (Fig. 1): from the anterior annulus fibrosus, across the nucleus pulposus, to the posterior annulus fibrosus.

### Illustration of division of discs into subregions

For each group (climbers and controls), signal mean and SD were calculated separately for each disc level. Within each disc level, values were computed both for the entire included disc region (all subregions combined within the analyzed midsagittal slices) and for each individual subregion. Calculations were performed using both a coarse subdivision of 5 subregions and a fine subdivision of 25 subregions. Group disc intensity profiles were summarized by the median of the subjects’ mean subregion signals, with interquartile ranges.

### Statistical analysis

Disc signals raw data were summarized by taking the mean of each subject, disc and subregion and entered in a generalized linear mixed model (GLMM) that was fitted to evaluate differences between climbers and controls across discs. The model included fixed effects for climber/control, disc, and their interaction, with subject as a random effect and a compound symmetry covariance structure to account for within-subject correlation. Degrees of freedom were estimated using the Kenward–Roger method. Least squares means were obtained, presenting mean and standard error, and group differences were assessed within each level of disc using sliced comparisons with 95% confidence limits. Results are presented both per disc level and per disc and subgroup level.

All analyses are two sided with alpha 0.05 using SAS 9.4 by SAS Institute Inc., Cary, NC, USA.

## Results

The resulting demographics and training habits of the participants are presented in Table 1, displaying a significant difference in weight and body mass index between climbers and controls.

**Table 1:** Demographics and training habits of participants

Parameter	Total (*n* = 30)	Climber (*n* = 15)	Control (*n* = 15)	*p*-value	Difference between groups Mean (95% CI)
Sex*					
Woman	16 (53.3%)	8 (53.3%)	8 (53.3%)		0.0 (-37.4; 37.4)
Man	14 (46.7%)	7 (46.7%)	7 (46.7%)	1.00	0.0 (-37.4; 37.4)
Age (years)	23.7 (2.5)	23.1 (3.2)	24.3 (1.5)	0.20	-1.27 (-3.2; 0.6)
Height (cm)	171.0 (7.3)	171.0 (7.9)	171.0 (7.0)	1.00	0.0 (-5.7; 5.7)
Weight (kg)	64.4 (9.6)	60.7 (10.5)	68.1 (7.1)	**0.04**	-7.3 (-14.2; -0.4)
BMI		20.6 (3)	23.2 (1.7)	**< 0.001**	-2.6 (− 4.0; − 1.2)
Maximum climbing level IRCRA	-	23.75 (5)	-		
Years climbing (climbers)/Years training (controls)		12 (5)	14 (7)		
Years Bouldering	-	11 (5)	-		
Yearly training volume					
< 400 h		2 (13)	8 (53)	0.05	
400–700 h		6 (40)	7 (47)		
> 700 h		7 (47)	-		

For categorical variables n (%) is presented. For continuous variables Mean (SD) is presented. Significant differences are marked in bold

*All participants reported gender in line with biological sex. The term sex is used throughout the report for simplicity

**Fig. 2:**
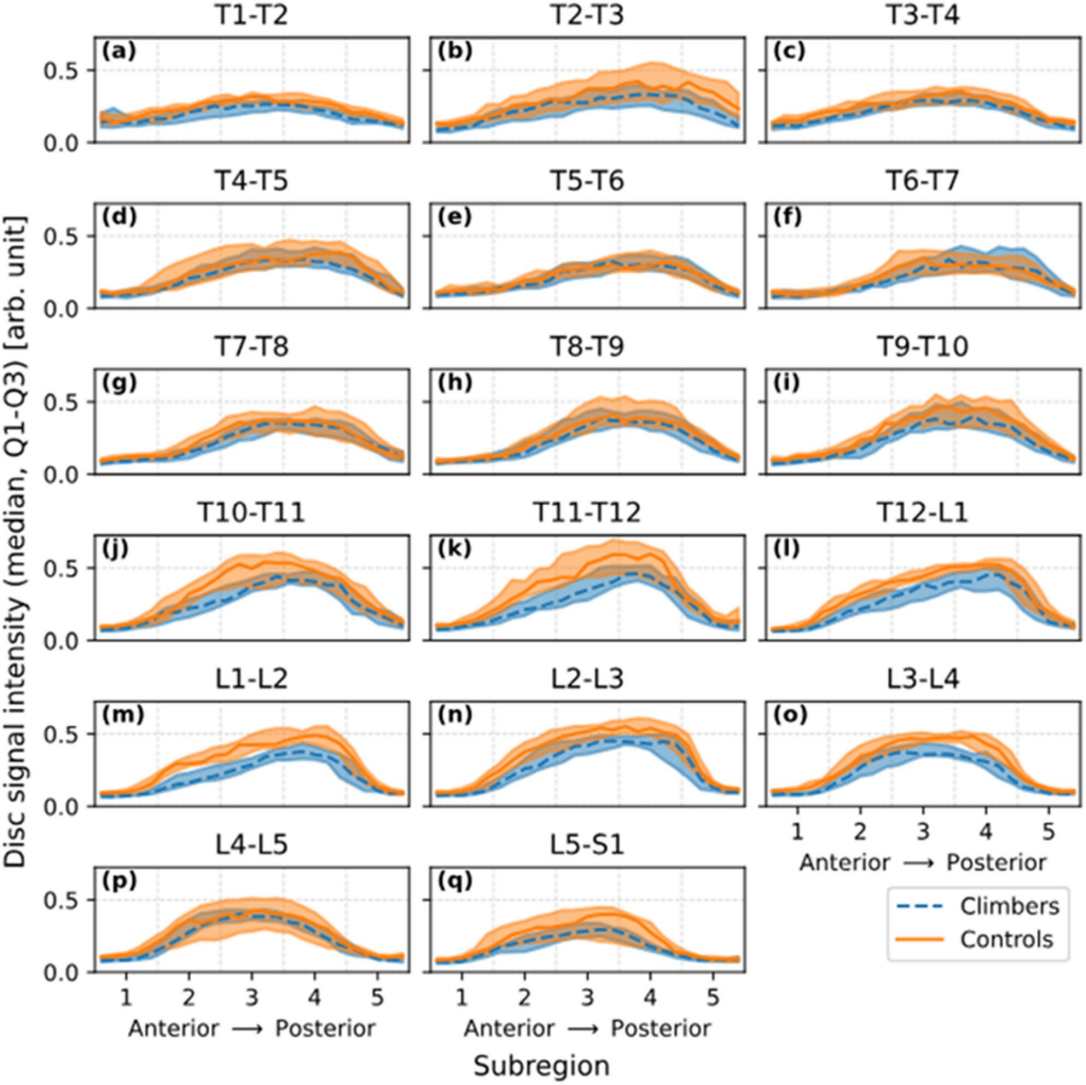
Mean signal across the discs across the thoraco-lumbar discs, T1-T2 to L5-S1 spinal levels ((**a**)-(**q**)) in climbers (dashed blue) and controls (orange). Curves show the median across subjects of their mean signal intensity values computed over 25 anterior–posterior disc subregions, normalized to the mean of the cerebrospinal fluid signal of each subject. Shaded bands indicate the interquartile range. The 25 finer subregions were further grouped into five broader regions (labeled 1–5), delineated by dashed vertical lines. As expected from disc composition, lower signal intensity means, reflecting reduced water content, were observed in the annulus fibrosus (subregions 1 and 5), while higher intensities were seen in the nucleus pulposus (subregions 2–4). Peak signal values typically occurred in subregion 3 but shifted toward subregion 4 in the thoracic spine, consistent with the more posterior position of the nucleus pulposus in this region. Clear visual group differences were also evident, climbers showed notably lower signal means than controls at levels T9–T10 through L3–L4, as well as at L5–S1

The data-driven MRI analysis clearly reflected the variation in disc water content (Fig. 2). Lower signal means, corresponding to reduced water content, were observed in the annulus fibrosus (subregions 1 and 5) compared to the hydrated nucleus pulposus (subregions 2–4). The highest signal means were generally observed in subregion 3 but shifted closer to subregion 4 in the thoracic spine, where the nucleus pulposus is more posteriorly positioned. Clear visual differences between climbers and controls are observed in levels T10-T11 to L3-L4 and L5-S1 where lower signal means are observed in the climbers. Statistical evaluation of these differences is included in the following results of the statistical analyses.

### Normalized T2-Weighted signal profiles across discs

Climbers and controls displayed significant differences in signal means within and between discs, depending on spinal level (Figs. 3 and 4; Table 2). Differences in disc characteristics between the groups were most pronounced at the thoracolumbar junction (*p* < 0.05 in T10-L4 discs and *p* < 0.01 T12-L2 discs; Fig. 3). More specifically, subregions 2–4, which represents the central region of the disc where the nucleus pulposus is located, showed markedly lower values in climbers than controls (Fig. 4).

**Table 2:** Disc signal mean (SE) by climbers and controls and mean difference with 95% CI per disc level and subregion

	Subregion														
	1			2			3			4			5		
Disc	Climbers Mean (SE)	Controls Mean (SE)	Difference Mean (95% CI)	Climbers Mean (SE)	Controls Mean (SE)	Difference Mean (95% CI)	Climbers Mean (SE)	Controls Mean (SE)	Difference Mean (95% CI)	Climbers Mean (SE)	Controls Mean (SE)	Difference Mean (95% CI)	Climbers Mean (SE)	Controls Mean (SE)	Difference Mean (95% CI)
T1-T2	0.16(0.01)	0.18(0.01)	0.02(-0.02,0.05)	0.21(0.02)	0.24(0.01)	0.03(-0.01,0.08)	0.26(0.02)	0.30(0.02)	0.04(-0.01,0.09)	0.23(0.02)	0.27(0.02)	0.05(-0.00,0.09)	0.16(0.01)	0.19(0.01)	0.04(0.00,0.07)
T2-T3	0.11(0.03)	0.12(0.02)	0.01(-0.15,0.17)	0.22(0.02)	0.25(0.02)	0.03(-0.03,0.09)	0.28(0.02)	0.34(0.03)	0.06(-0.02,0.14)	0.31(0.02)	0.39(0.04)	0.09(-0.01,0.18)	0.23(0.02)	0.32(0.04)	0.09(-0.01,0.20)
T3-T4	0.13(0.01)	0.17(0.01)	**0.03(0.01**,**0.06)**	0.22(0.01)	0.25(0.01)	0.03(-0.01,0.07)	0.29(0.02)	0.31(0.02)	0.02(-0.03,0.08)	0.28(0.02)	0.30(0.02)	0.02(-0.03,0.07)	0.16(0.01)	0.18(0.01)	0.02(-0.02,0.05)
T4-T5	0.12(0.01)	0.13(0.01)	0.01(-0.02,0.05)	0.21(0.02)	0.25(0.03)	0.04(-0.03,0.11)	0.32(0.02)	0.34(0.03)	0.03(-0.05,0.10)	0.34(0.02)	0.37(0.03)	0.03(-0.04,0.11)	0.22(0.02)	0.25(0.02)	0.02(-0.04,0.09)
T5-T6	0.12(0.01)	0.13(0.01)	0.01(-0.02,0.04)	0.17(0.01)	0.19(0.01)	0.02(-0.02,0.05)	0.28(0.02)	0.30(0.02)	0.02(-0.05,0.09)	0.31(0.02)	0.31(0.02)	-0.01(-0.07,0.06)	0.19(0.02)	0.20(0.02)	0.01(-0.04,0.06)
T6-T7	0.10(0.01)	0.11(0.01)	0.01(-0.01,0.03)	0.16(0.01)	0.20(0.02)	0.05(-0.00,0.10)	0.28(0.02)	0.31(0.03)	0.03(-0.04,0.10)	0.32(0.03)	0.31(0.02)	-0.01(-0.08,0.06)	0.21(0.02)	0.22(0.02)	0.01(-0.05,0.07)
T7-T8	0.10(0.01)	0.12(0.01)	0.02(0.00,0.04)	0.16(0.01)	0.21(0.02)	0.05(0.00,0.09)	0.31(0.03)	0.35(0.02)	0.04(-0.03,0.11)	0.33(0.02)	0.36(0.03)	0.04(-0.03,0.11)	0.21(0.02)	0.23(0.02)	0.02(-0.03,0.07)
T8-T9	0.10(0.01)	0.11(0.01)	0.01(-0.01,0.03)	0.19(0.03)	0.25(0.04)	0.05(-0.04,0.15)	0.35(0.03)	0.38(0.04)	0.03(-0.06,0.12)	0.36(0.02)	0.40(0.03)	0.04(-0.04,0.12)	0.20(0.02)	0.23(0.02)	0.03(-0.02,0.08)
T9-T10	0.10(0.01)	0.12(0.01)	0.03(0.01,0.04)	0.19(0.02)	0.25(0.02)	0.06(-0.00,0.12)	0.35(0.03)	0.41(0.03)	0.06(-0.02,0.14)	0.38(0.03)	0.42(0.04)	0.04(-0.05,0.13)	0.19(0.01)	0.23(0.02)	0.04(-0.01,0.09)
T10-T11	0.10(0.01)	0.13(0.01)	0.03(0.00,0.06)	0.22(0.02)	0.33(0.03)	**0.11(0.03**,**0.18)**	0.37(0.02)	0.48(0.03)	**0.11(0.03**,**0.19)**	0.41(0.02)	0.46(0.03)	0.06(-0.02,0.13)	0.21(0.02)	0.24(0.02)	0.03(-0.03,0.09)
T11-T12	0.11(0.01)	0.17(0.03)	0.07(-0.01,0.14)	0.23(0.02)	0.39(0.05)	**0.16(0.04**,**0.28)**	0.37(0.03)	0.54(0.05)	**0.17(0.05**,**0.29)**	0.42(0.02)	0.52(0.04)	**0.10(0.02**,**0.19)**	0.18(0.01)	0.22(0.02)	0.04(-0.01,0.09)
T12-L1	0.09(0.01)	0.13(0.02)	**0.04(0.01**,**0.08)**	0.23(0.01)	0.33(0.02)	**0.10(0.04**,**0.16)**	0.35(0.02)	0.46(0.03)	**0.12(0.04**,**0.19)**	0.41(0.02)	0.51(0.02)	**0.10(0.04**,**0.16)**	0.20(0.02)	0.25(0.03)	0.05(-0.02,0.12)
L1-L2	0.09(0.01)	0.12(0.01)	**0.04(0.02**,**0.05)**	0.19(0.01)	0.31(0.02)	**0.12(0.07**,**0.16)**	0.31(0.02)	0.44(0.02)	**0.13(0.06**,**0.19)**	0.36(0.02)	0.47(0.02)	**0.12(0.05**,**0.19)**	0.15(0.01)	0.19(0.01)	**0.05(0.01**,**0.08)**
L2-L3	0.10(0.01)	0.13(0.01)	**0.03(0.01**,**0.05)**	0.27(0.02)	0.37(0.02)	**0.10(0.04**,**0.16)**	0.43(0.02)	0.52(0.03)	**0.09(0.01**,**0.18)**	0.44(0.02)	0.54(0.03)	**0.10(0.03**,**0.17)**	0.18(0.01)	0.22(0.01)	**0.04(0.01**,**0.08)**
L3-L4	0.09(0.00)	0.13(0.01)	**0.04(0.02**,**0.06)**	0.26(0.02)	0.36(0.03)	**0.10(0.03**,**0.17)**	0.38(0.02)	0.49(0.03)	**0.11(0.04**,**0.18)**	0.31(0.02)	0.42(0.03)	**0.12(0.04**,**0.19)**	0.11(0.01)	0.14(0.01)	**0.03(0.01**,**0.05)**
L4-L5	0.10(0.01)	0.14(0.01)	**0.04(0.02**,**0.06)**	0.29(0.02)	0.30(0.03)	0.01(-0.06,0.08)	0.39(0.02)	0.38(0.04)	-0.00(-0.09,0.08)	0.29(0.02)	0.31(0.03)	0.01(-0.06,0.09)	0.12(0.01)	0.13(0.01)	0.01(-0.01,0.03)
L5-S	0.09(0.01)	0.12(0.01)	**0.03(0.01**,**0.05)**	0.21(0.02)	0.28(0.03)	0.07(-0.00,0.14)	0.27(0.02)	0.34(0.04)	0.07(-0.02,0.15)	0.19(0.01)	0.26(0.03)	0.07(0.00,0.13)	0.09(0.00)	0.10(0.00)	0.01(-0.00,0.02)

Significant differences are marked in bold

### Forest plot of T2-weighted signal mean differences across discs in climbers and controls

**Fig. 3:**
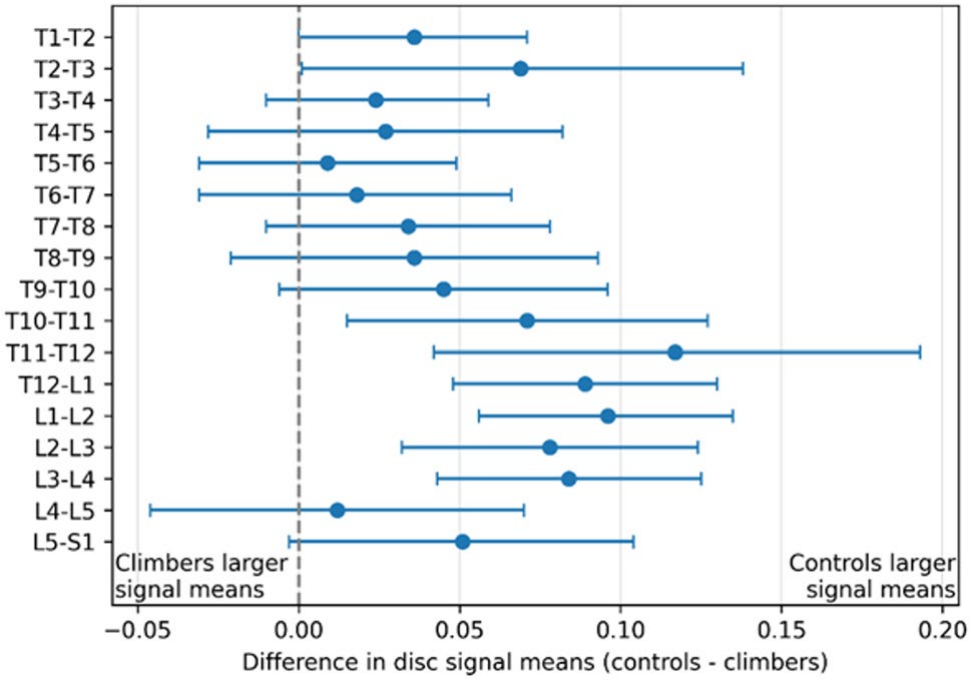
Forest plot, visualizing the difference in disc signal means at the midsagittal regions in the discs of the thoraco-lumbar spine for climbers and controls. Each point represents the mean difference between climbers and controls, with horizontal bars showing the 95% confidence interval. Values positioned to the left or right of the vertical zero line indicate higher signal intensity mean in climbers or controls, respectively. Differences are considered statistically significant when the confidence interval does not cross zero. Lower values in the climbers, especially at the thoracolumbar junction, indicate early signs of disc degeneration, which was more pronounced

### Forest plot of T2-weighted signal mean differences across disc subregions in climbers and controls

**Fig. 4:**
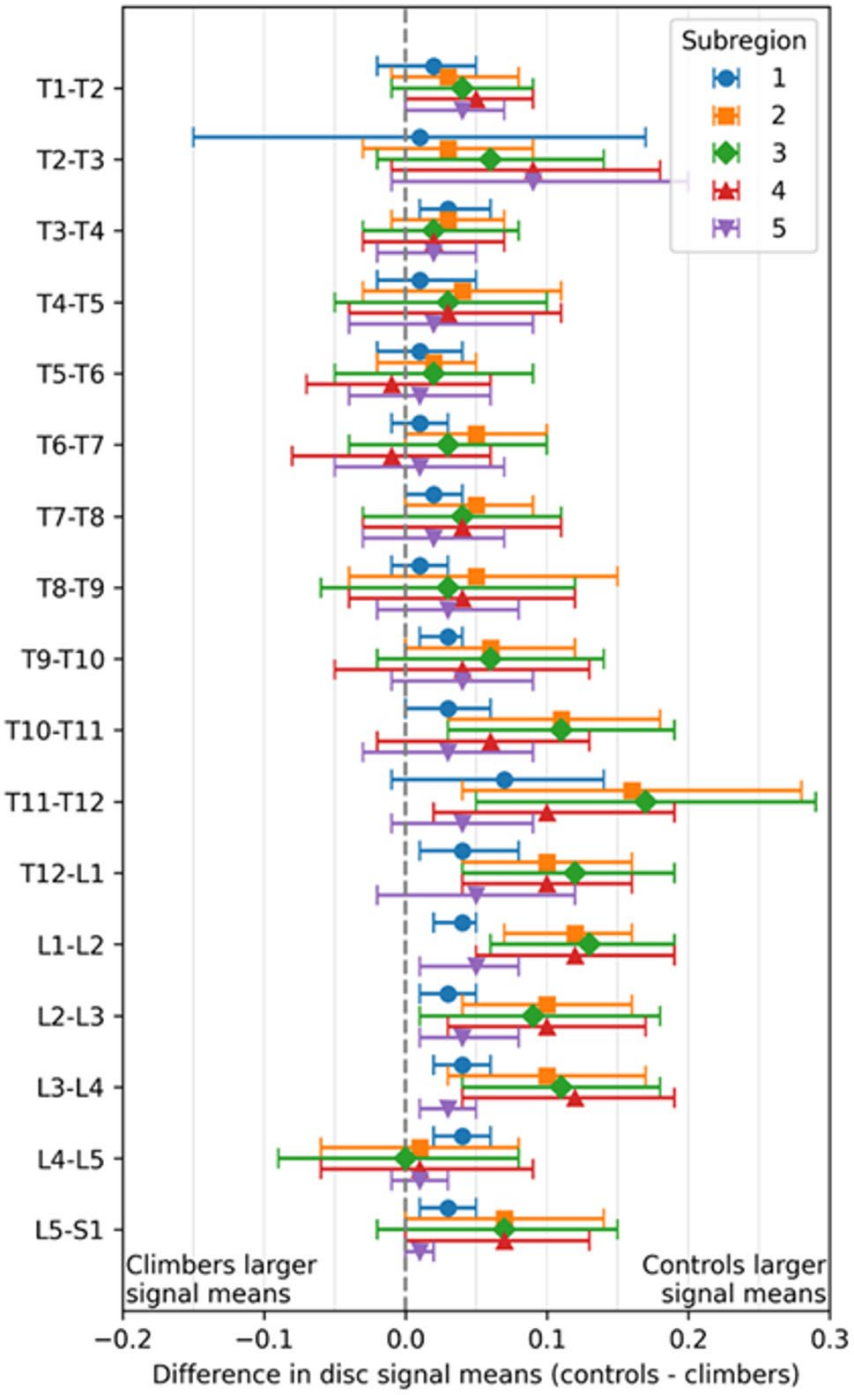
Forest plot, visualizing the difference in signal means at different subregions of the discs (1: anterior annulus fibrosus, 2–3: nucleus pulposus, 5: posterior annulus fibrosus) of the thoraco-lumbar spine for climbers and controls. Each point represents the mean difference between climbers and controls, with horizontal bars showing the 95% confidence interval. Values positioned to the left or right of the vertical zero line indicate higher signal intensity mean in climbers or controls, respectively. Differences are considered statistically significant when the confidence interval does not cross zero. Markedly lower values in the climbers at subregion 2–4 indicate reduced hydration in the nucleus pulposus, which was most pronounced at the thoracolumbar junction

## Discussion

This MRI study did quantitatively establish lower disc signal intensity in the elite climbers compared with controls, indicating dehydration of the discs in these individuals. The disc dehydration was most pronounced in the thoracolumbar junction and in the central region of the disc representing the nucleus pulposus of the climbers. Notably, these group differences were evident even though the control group reported a relatively high level of physical activity, supporting the hypothesis that climbing, like numerous other sports at an elite level, may accelerate the degenerative process in the spine [26, 37, 38].

Although quantitative MRI studies on the spine in elite athletes are sparse, our findings of reduced T2-weighted signal intensity in the thoracolumbar discs of climbers are consistent with observations in other high-load sports. Lagerstrand et al. [27] reported lower T2-values and altered histogram distribution in young elite skiers compared to controls, particularly in lumbar regions exposed to repetitive stress, indicating early degenerative changes. Similarly, a study of elite rowers [39], using T2* mapping, also found significantly lower values, and higher Pfirrmann grades, compared to non-athletes. Collectively, these findings suggest that intensive, sport-specific loading accelerates disc tissue adaptation and may predispose athletes to early degeneration, reinforcing the value of quantitative MRI biomarkers for early detection.

In elite climbing, repeated landings inherent to bouldering are considered the primary mechanism for recurrent loading of the spine. These are the same loads that ex vivo studies have shown to accelerate the degenerative process in both the intervertebral discs and endplates [4]. During climbing, sport-specific movements also involve a high degree of axial rotation, lateral flexion, sagittal plane flexion, as well as episodes of hyperextension, which collectively may also contribute to increased mechanical stress on the spine.

While our study primarily focused on the mechanical loading associated with jumping in bouldering as a potential driver of changes in the disc nucleus, it is important to recognize that additional factors may contribute to these observations. Climbers are frequently exposed to strength training, which, if performed with improper technique or high intensity, may induce similar mechanical stresses on the spine and intervertebral discs. Furthermore, overhead arm activity and sport-specific postural adaptations can create muscle adaptions, shortening of certain muscle groups, and weakening of others, potentially influencing spinal biomechanics and the intervertebral disc nucleus. Previous studies have highlighted these effects. Kiełt et al. [40] reported characteristic postural adaptations in climbers, while Förster et al. [5] demonstrated correlations between climbing ability, sagittal spinal curvature changes, and pectoral muscle contractures, all of which may indirectly affect disc health.

In a previous study, on the same dataset, conventional MRI classification systems were used to characterize the spinal tissues (Pfirrmann classification, Modic classification, Endplate Defect Score, and categorical grading of apophyseal injuries). In this study no significant group differences could be established [17]. In contrast, data-driven analysis with objective tissue characteristics could here reveal accelerated disc degeneration in the climbers, underscoring the value of quantitative MRI to detect relevant changes that conventional methods may overlook.

Conventional MRI classification systems are designed to characterize spinal changes in the general population. Such image analyses are largely focused on signs of degeneration associated with aging [12]. Still, such have been used to identify signs of injury in young athletes with questionable transferability [41–47]. The use of data-driven MRI methods addresses these limitations by eliminating reliance on observer-based visual grading, instead providing continuous variables with multiple data points from each disc. The sensitive and specific MRI biomarker presented herein has previously been used in studies of other groups of athletes with high axial loads [7, 8] and show, in consensus with the present work, promise for broader use in sports medicine [27–29, 48].

Though sport participation has many benefits, and should be promoted, elite level participation is plagued by the risk of multiple overuse-related changes. Identifying and highlighting the sport specific risks are key to develop effective injury prevention programs and to guide training regimes [49]. In bouldering, there are techniques to decrease the number and height of jumps and among these are designated decent routes for down climbing rather than jumping down.

Protective and strengthening training programs also play a critical role in supporting athletic performance and long-term musculoskeletal health. Based on our findings, we suggest that spinal MRI in climbers should primarily be reserved for athletes reporting pain or other spinal symptoms, rather than as part of routine preventive screening. This approach allows clinicians to target imaging resources effectively while identifying early intervertebral disc changes in symptomatic individuals. Preventive programs may still focus on technique optimization, balanced strength training, and posture correction to reduce biomechanical stress on the spine, potentially lowering the risk of disc degeneration. Regular monitoring through clinical assessment, rather than imaging alone, is recommended to guide individualized interventions and ensure timely evaluation when symptoms arise.

### Limitations

This study had some limitations. The study group is limited in size due to the available number of elite climbers in Sweden. Another potential limitation could be selection bias. Only climbers with moderate spinal abnormalities are represented, as individuals with severe back pain and/or tissue degeneration most probably need to quit the sport. It can also be noted that spinal sagittal alignment, which was not evaluated, could possibly pose a selection bias that could influence the results. Lastly, since the specific loading conditions experienced by the athletes could not in any reliable way be documented, we cannot determine which specific loads may have contributed to the observed results.

## Conclusion

The present findings revealed early signs of degenerative changes in the thoracic and lumbar spine in elite level climbers. Especially at the thoracolumbar level and the central parts of the discs significant dehydration was demonstrated. Further, the study confirms that the use of quantitative MRI biomarkers seems to be a promising tool in the evaluation of sport-related spinal changes.

## Supplementary Information

Supplementary Material 1.

## Data Availability

The data that support the findings of this study are available from the corresponding author upon reasonable request.

## Declarations

Informed written consent was obtained from all participants.

## Abbreviations

IRCRA: International Rock Climbing Research Association
MRI: Magnetic Resonance Imaging
SD: Standard Deviation
