# Supplementary material for: Elite rock-climbers exhibit early signs of degenerative spinal changes; enhanced analysis using quantitative MRI
Source: BMC Sports Sci Med Rehabil. 2026 Feb 13;18:143. doi: 10.1186/s13102-026-01579-y (PMC13005541; doi:10.1186/s13102-026-01579-y)
Supplement: Supplementary file 1 — Supplementary Material 1. [file 13102_2026_1579_MOESM1_ESM.docx]

**Questionnaire
Baseline data, climbing performance and training habits**

The following questions are designed to provide information regarding your climbing performance and training habits

Personal ID-number:

How tall are you (cm)?

How much do you weigh?

How many years have you been climbing in total?

How many years have you been bouldering in total?

How many years have been competing in climbing?

**1. Self-assessed redpoint ability in climbing**

| What is **the most difficult grade** at which you have completed at least three different routes (redpoint/repeated attempts) **over the last three months?** Use french sport grading  *(6a+, 6b…) for sport climbing and the Font-scale for bouldering (6A, 6A+…).*  *Eg. If you have climbed one 7a+, one 7b+ and one 7c+ your most difficult route will be 7a+* | **Indoor**  Boulder:  Sport: | **Outdoors**  Boulder:  Sport: |
| --- | --- | --- |

**2. Self-assessed redpoint ability redpoint**

| What is **the most difficult grade** at which you have completed at least three different routes (redpoint/repeated attempts) **over the last twelve months?**  Use french sport grading  *(6a+, 6b…) for sport climbing and the Font-scale for bouldering (6A, 6A+…).*  *Eg. If you have climbed one 7a+, one 7b+ and one 7c+ your most difficult route will be 7a+* | **Indoor**  Boulder:  Rep: | **Outdoors**  Boulder:  Rep: |
| --- | --- | --- |

What is your total annual training volume in climbing?

- Under 400h
- 401 – 549h
- 550 – 700h
- Over 700h

What is your annual training volume in bouldering?

- Under 400h
- 401 – 549h
- 550 – 700h
- Over 700h

What was your total annual training volume in climbing between 10-15 years of age?

- Under 400h
- 401 – 549h
- 550 – 700h
- Over 700h

What was your total annual training volume in climbing between 16-20 years of age?

- Under 400h
- 401 – 549h
- 550 – 700h
- Over 700h

When bouldering – how often do you decend from the route by jumping down on the mat?

1. Always
2. Almost always
3. Most often
4. About half of the times
5. Rarely
6. Almost never
7. Never
